# *Rickettsia felis* Infection among Humans, Bangladesh, 2012–2013

**DOI:** 10.3201/eid2108.150328

**Published:** 2015-08

**Authors:** Faria Ferdouse, Muhammad Akram Hossain, Shyamal Kumar Paul, Salma Ahmed, Md Chand Mahmud, Rajib Ahmed, A.K.M. Fazlul Haque, M. Nur-a-Alam Khan, Souvik Ghosh, Noriko Urushibara, Nobumichi Kobayashi

**Affiliations:** Mymensingh Medical College, Mymensingh, Bangladesh (F. Ferdouse, M.A. Hossain, S.K. Paul, S. Ahmed, M.C. Mahmud, R. Ahmed, A.K.M.F. Haque, M.N.A. Khan);; Sapporo Medical University School of Medicine, Sapporo, Japan (S. Ghosh, N. Urushibara, N. Kobayashi);; Ross University School of Veterinary Medicine, St. Kitts, West Indies (S. Ghosh)

**Keywords:** Rickettsia felis, febrile illness, Bangladesh, 17kDa antigen, 16S rRNA, gltA, ompA, ompB, rickettsia, bacteria

**To the Editor:** *Rickettsia felis*, which belongs to the spotted fever group of rickettsiae, causes febrile illness in humans. The main vector of this bacterium is the cat flea (*Ctenocephalides felis*). Since publication of reports of *R. felis* as a putative pathogen of humans in the United States in 1994, *R. felis* infection in humans worldwide has been increasingly described, especially in the Americas, Europe, Africa, and eastern Asia (*1*,*2*). *R. felis* infection is common among febrile patients (≈15%) in tropical Africa (*3*) and among apparently healthy persons in eastern coastal provinces of China (*4*). However, little is known about prevalence of *R. felis* infection of humans in southern Asia, although 3 serologically diagnosed cases in Sri Lanka have been described (*5*) and *R. felis* has been detected in rodent fleas in Afghanistan (*6*). Hence, we conducted a cross-sectional study in Bangladesh to explore the presence of rickettsial pathogens among patients with fever of unknown origin.

Study participants were 150 patients at Mymensingh Medical College (MMC) hospital in Mymensingh, north-central Bangladesh, from July 2012 through January 2014, and 30 healthy control participants from the staff at the same college. Selected patients met the following criteria: 1) fever (axillary temperature >37.5°C) for >15 days that did not respond to common antimicrobial drug therapy; 2) any additional clinical features including headache, rash, lymphadenopathy, myalgia, and eschars on skin; and 3) titer according to the Weil-Felix test (antibodies against any of 3 *Proteus* antigens) of >1:80. Patients with evident cause of fever (e.g., malaria diagnosed by blood smear or immunochromatography) were excluded from the study. This research was approved by the college institutional review board, and informed consent was obtained from patients (or guardians) and healthy controls before their entry into the study.

Venous blood samples were aseptically collected from the patients, and DNA was extracted by conventional method by using proteinase K and sodium dodecyl sulfate. Nested PCR selective for the 17-kDa antigen gene was used to screen for rickettsiae according to the method described previously (*7*); ≈100 ng of DNA in a 50-μL reaction mixture was used. For each PCR, a negative control (water) was included and utmost care was taken to avoid contamination. Among the 150 samples tested, results were positive with a 232-bp amplified product for 69 (46%) and negative for all controls. 

PCR products from 20 samples were randomly selected for sequence analysis. All nucleotide sequences from the 17-kDa antigen gene (186-bp) were identical to that of reference strain *R. felis* URRWXCa12 (GenBank accession no. CP000053). Among all 17-kDa–positive samples, positivity was further confirmed by PCR detection of the *R. felis* 16S rRNA gene and *gltA* in 95% and 75% of samples, respectively. Partial 16S rRNA gene sequences (305-bp) from 12 samples were 100% or 99% (10 and 2 samples, respectively) identical to that of *R. felis* URRWXCa12. The complete open reading frames of *ompA* (1773-bp), partial *ompB* (413-bp), and *gltA* (611-bp) sequences determined for 3, 3, and 5 samples, respectively, were also identical to those of *R. felis* URRWXCa12. The 5 gene sequences were determined for samples from 3 patients (2-year-old girl, 8-year-old boy, 17-year-old boy). The 5 gene sequences from the 2-year-old girl (strain Ric-MMC7) and 2 partial sequences of 16S rRNA (Ric-MMC71 and Ric-MMC133) were deposited in GenBank under accession nos. KP318088–KP318094.

According to PCR, the positivity rate for the *R. felis* 17-kDa antigen gene was higher among male (54%, 40/74) than among female (38%, 29/76) patients and higher among patients in young and old age groups (0–15 years, 57%; 45–60 years, 62%) than among patients in other age groups (15–30 years, 41%; 30–45 years, 44%). During the study period, rates of *R. felis* positivity were highest during the late rainy season of 2012 (September [59%] and October [52%]) and lowest (0%) from December 2012 through April 2013 (Figure). The rate was significantly higher among farmers (76%, 13/17) than among persons of other occupations (e.g., housewives, teachers, students) (42%, 56/133); p = 0.016. Among the 69 rickettsiae-positive patients, headache and myalgia were reported by 29 (42%) and 17 (25%), respectively, whereas rash was detected in only 2 (3%) patients, both of whom were female.

**Figure:**
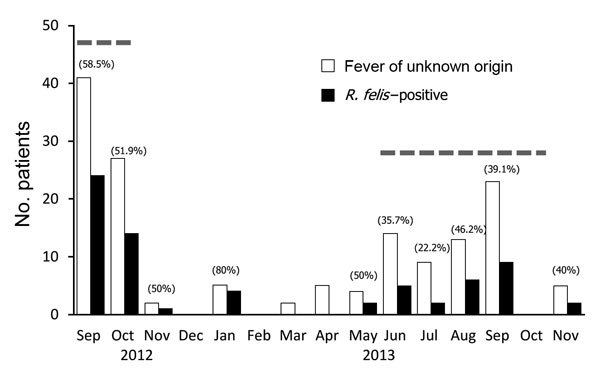
Number of patients with fever of unknown origin and *Rickettsia felis–*positive cases in the Mymensingh Medical College hospital, Bangladesh, 2012–2013. Numbers in parentheses indicate rates of *R. felis* positivity for each month; dashed lines indicate monsoon season (June–October).

This study demonstrated *R. felis* infection in patients in Bangladesh with unidentified febrile illness. The high prevalence (46%) of *R. felis* infection suggests that this infection is endemic to the north-central area of this country and might be associated with contact between humans of low socioeconomic status and the large number of stray cats and dogs. In contrast, the number of genetically confirmed cases of *R. felis* infection in humans reported to date in China, Taiwan, Thailand, and Laos have been very few (*1*,*2*,*4*,*8*–*10*), although widespread presence of this bacterium in cat fleas has been documented. For further confirmation of spread of this infectious disease, the prevalence of *R. felis* infections among humans, vectors, and reservoirs in other areas in Bangladesh and in other countries in southern Asia should be investigated. 
